# Microglial Phenotypic Transition: Signaling Pathways and Influencing Modulators Involved in Regulation in Central Nervous System Diseases

**DOI:** 10.3389/fncel.2021.736310

**Published:** 2021-09-14

**Authors:** Jiaxin Li, Xinyu Shui, Ruizheng Sun, Lily Wan, Boxin Zhang, Bo Xiao, Zhaohui Luo

**Affiliations:** ^1^Department of Neurology, Xiangya Hospital, Central South University, Changsha, China; ^2^Xiangya School of Medicine, Central South University, Changsha, China

**Keywords:** neuroinflammation, polarization, cytokines, iron channels, receptors, oxidative stress reaction, microRNA

## Abstract

Microglia are macrophages that reside in the central nervous system (CNS) and belong to the innate immune system. Moreover, they are crucially involved in CNS development, maturation, and aging; further, they are closely associated with neurons. In normal conditions, microglia remain in a static state. Upon trauma or lesion occurrence, microglia can be activated and subsequently polarized into the pro-inflammatory or anti-inflammatory phenotype. The phenotypic transition is regulated by numerous modulators. This review focus on the literature regarding the modulators and signaling pathways involved in regulating the microglial phenotypic transition, which are rarely mentioned in other reviews. Hence, this review provides molecular insights into the microglial phenotypic transition, which could be a potential therapeutic target for neuroinflammation.

## Introduction

Microglia are tissue macrophages present in the central nervous system (CNS) that account for 5–20% of all glial cells present in the adult mammalian brain. Microglia are derived from erythroid myeloid precursors in the embryonic yolk sac and are distributed within the embryonic mouse brain (Ginhoux et al., 2010; Gomez Perdiguero et al., 2013). During early embryonic development, microglia are very close to neurons and other glial cells; further, they are crucially involved in CNS development and homeostasis (Schafer and Stevens, 2015). Microglia are associated with the interaction between adult CNS neurons and innate immune cells; moreover, they are associated with aging-related neurological diseases (Malm et al., 2015). There has been a gradual recent increase in the immunological potential of microglia. In normal conditions, microglia interact with neurons to maintain neuronal functions, including plasticity. Concomitantly, healthy neurons maintain microglia at rest using membrane-binding signals, including CD200 and CX3CL1, as well as secretory factors, including neurotransmitters and neurotrophic proteins (Raivich, 2005; Hanisch and Kettenmann, 2007). There are numerous receptors for microglia, including toll-like receptors (TLRs), receptors for advanced glycation end products, scavenger receptors, neurotransmitter-binding receptors (e.g., glutamate, γ-aminobutyric acid, dopamine, and epinephrine), cytokines, chemokines, and purines. Microglia are activated by sensing danger signals through these receptors (Joshi and Singh, 2018).

Previous studies have suggested that activated microglia promote neuroinflammation occurrence and development; moreover, they aggravate neuronal injury (Ouchi et al., 2005; Block et al., 2007). With the continuous development of the neuroscience field, researchers have discovered that microglia not only play a role in injury and destruction, but may also promote tissue repair. Different phenotypes of microglia have their own physiological functions (Table 1). Among the functions of microglia, the most important one is undoubtedly neuroinflammation. Microglia with different phenotypes either promote neuroinflammation and aggravate neuronal damage, or inhibit neuroinflammation and promote neuron repair and regeneration (Mantovani et al., 2004; Fenn et al., 2012; Varnum and Ikezu, 2012; Chhor et al., 2013; Pena-Altamira et al., 2016).

**TABLE 1:** Stimulations, markers, and functions of different subtypes of microglia.

**Phenotype**	**Stimulations**	**Marker**	**Function**	**References**
M1	LPS, IFN-γ, TNFα	iNOS, Cox-2, CXCL9, CXCL10, TNF-α, IL-2, IL-6, IL-1β	Cytotoxic properties	Burton et al., 2011; Varnum and Ikezu, 2012; Chhor et al., 2013
M2a	IL-4, IL-13	Arg-1, CD206, Ym1, Fizz1, IGF-1	Nerve repair and regeneration	Chhor et al., 2013
M2b	LPS, IL-1β, TNFα	IL-1Rn, SOCS3	Immunoregulatory	Chhor et al., 2013
M2c	IL-10, TGF-β	IL-4Rα, CXCL13, SOCS3	Immunoregulatory	David and Kroner, 2011; Fenn et al., 2012; Hu et al., 2012

*Arg-1, arginase-1; Cox-2, cyclooxygenase-2; CXCL, chemokine (C-X-C motif) ligand; IFN-γ, interferon-γ; IGF-1, insulin growth factor-1; IL, interleukin; LPS, lipopolysaccharide; SOCS3, suppressor of cytokine signaling 3.*

Neuroinflammation is associated with the occurrence and development of numerous CNS diseases, including neurodegenerative diseases (including Parkinson’s disease and Alzheimer’s disease), stroke, multiple sclerosis (MS), and epilepsy. The phenotype transition of microglia is closely associated with the prognosis of these CNS diseases. In Parkinson’s disease (PD), misfolded α-synuclein, pathogens, or environmental toxins can cause the activation of microglia directly or indirectly, causing the upregulation of pro-inflammatory cytokines while the polarization of M2 microglia has hardly been reported in PD (Blandini, 2013; Tang and Le, 2016). Promoting the polarization of microglia to the M1 phenotype can aggravate the death of dopaminergic neurons, which can exacerbate the symptoms of Parkinson’s disease (Du et al., 2018). On the contrary, the polarization of M2 microglia improves the learning and memory deficits of patients (Che et al., 2018; Hou et al., 2019). Accumulation of amyloid-β (Aβ) is one of the pathological features of Alzheimer’s disease (AD), which can lead to the impairment of cognitive function. Endogenous stimulation factors, such as reactive oxygen species (ROS), inflammatory factors, and Aβ stimulate microglia to turn into M1 phenotype persistently, promoting neuroinflammatory response and ultimately leading to irreversible neuron loss (Deczkowska et al., 2018; Sepulcre et al., 2018). M2 microglia can promote the clearance of Aβ through phagocytosis, and reduce the brain inflammation and toxic effect of Aβ (Ren et al., 2020). After ischemic stroke, microglia are activated and polarized into pro-inflammatory M1 type or anti-inflammatory M2 type, and participate in the secondary brain injury or nervous tissue repair by producing different immunomodulatory molecules (Raivich, 2005; Jiang et al., 2020; Zhang L. et al., 2020). Reducing the polarization of M1 microglia can decrease the inflammatory reaction and the area of infarcted brain tissue (Chen Y. J. et al., 2018). The identification of the brain slices of patients with progressive MS death reveals that the myelin sheath of the chronically active lesions is completely shedding, and myelin degradation products can be detected in the cytoplasm of microglia at the lesion edge (Jackle et al., 2020). Compared with chronic inactive lesions, M1 microglia tend to accumulate around the chronic active lesions, indicating that the chronic active lesions in progressive MS are mainly driven by microglia, with M1 polarization dominated lesions (Jackle et al., 2020). During the recovery process of MS, M2 type microglia assist in the regeneration of myelin sheath, remove the injured myelin sheath fragments effectively, release nutritional support factors, and relieve the symptoms of MS (Napoli and Neumann, 2010; Bao et al., 2019). Phenotypic transition of microglia also can be observed in epilepsy (Musto et al., 2011; Benson et al., 2015). M1 microglia significantly increased at the acute phase of epilepsy and continued to maintain a high level during the incubation period, while the M2 microglia decreased significantly and then gradually increased to reach a peak at 20 days after status epilepticus (Li et al., 2017). Increasing the proportion of M2 microglia can reduce the frequency, duration, and severity of spontaneous seizures, and improving the outcome of epilepsy (Li et al., 2017).

Hence, regulating microglial phenotype transition has gradually become a potential therapeutic strategy for these CNS diseases (Vezzani and Granata, 2005; Tang and Le, 2016; Miao et al., 2019; Ma et al., 2020; Zhang et al., 2010). This review focuses on the various signaling pathways and regulatory modulators involved in regulating microglial phenotypic transitions in CNS disease.

## Status and Controversy of Classification of Microglia

Microglia are macrophages residing in the CNS and are crucial components of the neuronal microenvironment (Sato, 2015). As a part of innate immunity, microglia form the first line of defense in neuroimmunology upon occurrence of injury or disease (Block and Hong, 2005; Glass et al., 2010). Like peripheral macrophages, microglia have two activation pathways; specifically, the classic M1 and alternative M2 pathways, which can be further divided into the M2a/b/c types after polarization. Polarized microglia cells are divided into various subtypes which have different inflammatory features according to the expression of characteristic markers; additionally, their polarization direction depends on different stimulations (Table 1). In general, M1 microglia represent a pro-inflammatory phenotype, while M2 microglia represent an anti-inflammatory phenotype.

However, with the development of neuroscience, the traditional M1/M2 classification has been questioned. Researchers hold different views on the accurate classification method for microglia. It is suggested that M1/M2 characterization is not adequate to define the inflammatory profile of microglia because microglia exhibit mixed phenotypes after polarization. In this case, characterizing the microglia by neuroinflammatory sequelae are more suitable (Rosi, 2016). It has also been pointed out that microglia exhibit unique phenotypes that express specific biomarkers in different CNS diseases states due to different microenvironments. The activation profile of microglia is not black and white, but a gray scale, depending on the conditions (Bachiller et al., 2018). Recently, a broad array of activation profiles and phenotypes has been identified, especially in the neurodegenerative disease (De Biase et al., 2017; Keren-Shaul et al., 2017).

In our view, the classification methods are so diverse and complex that it is difficult to have a unified and widely accepted standard. The traditional M1/M2 classification method does have some limitations, but it is still worthy of reference. More importantly, compared with other classification methods, the M1/M2 classification is more suitable for our review. Although it is true that microglia exhibit mixed phenotypes after polarization, there is no mature and responsible microglia classification based on neuroinflammatory sequelae (Tang and Le, 2016; Geloso et al., 2017; Qin et al., 2019). Therefore, this approach is rarely used in neuroscience field. On the contrary, despite the above limitations, M1/M2 classification is still widely used due to the existence of objective biomarkers that can identify microglia of different phenotypes (Table 1). In this review, we focus not on the whole polarized state of microglia, but on the proportion of M1/M2 microglia in the population, particularly on the transition between M1/M2 microglia. So, we are more interested in the process of microglia phenotypic transition than in the symptoms of neuroinflammatory sequelae. Besides, the transition between M1 and M2 microglia represents the ultimate neuroinflammatory sequelae to some extent. Furthermore, we focus on the modulators and signal pathways in microglia phenotypic transition. To find a general pattern in regulation of microglia phenotypic transition, our review is not classified by diseases, but concentrated on modulators and signal pathways. It is also unreasonable to classify microglia according to the unique phenotypes in different CNS diseases in this paper. Most importantly, despite the limitations of M1/M2 classification, it is still widely used in the current experimental articles of microglia phenotypic transition because it takes time for the classification to be updated and there is no better unified standard for microglia classification so far. Rather than being a limited classification method, “M1 microglia” and “M2 microglia” seem to represent two opposite manifestations of neuroinflammation (pro-inflammatory and anti-inflammatory) in a broad sense, which can lead to completely different changes in the process of CNS diseases. Collectively, this review is based on the M1/M2 classification of microglia, which is still widely used by researchers in neuroscience field.

**FIGURE 1:**
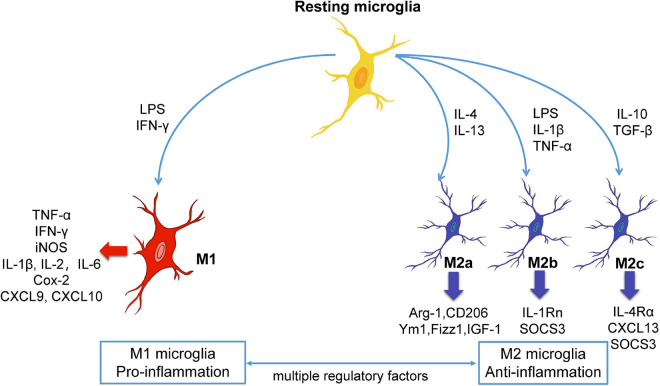
The polarization and phenotypic transition of microglia. Microglia are remaining stationary under normal state, and different stimulators can induce the resting microglia to become different phenotypes. In general, M1 microglia are pro-inflammatory phenotype while M2 microglia are anti-inflammatory phenotype. M1 or M2 microglia can release different substances, and the microglia of the two phenotypes can be converted to each other under certain conditions. Arg-1, arginase-1; Cox-2, cyclooxygenase-2; CXCL, chemokine (C-X-C motif) ligand; IFN-γ, interferon-γ; IGF-1, insulin growth factor-1; IL, interleukin; LPS, lipopolysaccharide; SOCS3, suppressor of cytokine signaling, TNF-α, tumor necrosis factor-α.

Regarding neuroinflammation, M1 and M2 microglia play diametrically opposite roles. M1 cells, which activated by lipopolysaccharide (LPS) and interferon-γ (IFN-γ), secrete IL-1β, IL-2, IFN-γ, CXCL9, CXCL10, iNOS, COX-2, and other pro-inflammatory cell factors with neurotoxic effects (Fenn et al., 2012; Varnum and Ikezu, 2012; Chhor et al., 2013). Contrastingly, M2 microglia, which activated by IL-4, IL-13, or IL-10, secrete brain-derived neurotrophic factor and transforming growth factor-β, as well as anti-inflammatory cytokines, including Ym1, Fizz1, IL-1 and IL-4, which block inflammatory reactions, promote repair and regeneration, and exert neuroprotective effect (Mantovani et al., 2004; Pena-Altamira et al., 2016; Figure 1). In CNS diseases, there is often an imbalance in the ratio of M1/M2 microglia, which accelerates the progression or deterioration of the disease. Several reviews describe the different phenotypes and characteristics of microglia in CNS disease (Ransohoff and El Khoury, 2015; Bachiller et al., 2018). Studies also have suggested that intervention involved in the transition between M1/M2 microglia has positive significance for the treatment of CNS diseases (Tang and Le, 2016; Zhang X. et al., 2020). However, the modulators and signaling pathways involved in the microglia phenotypic transition have not been reviewed and summarized at present. Novelly, we will summarize and conclude the modulators and signaling pathways involved in the microglia phenotypic transition in order to provide molecular insights into the microglial phenotypic transition in this paper.

## Modulators Influencing Microglial Phenotypic Transition

Various modulators can regulate M1/M2 microglia phenotypic transition. A potential therapeutic strategy involves promoting microglial transition to the protective M2 phenotype and inhibiting over-activated toxic M1 microglia. Next, we classify the modulators that affect microglial phenotypic transition and review the possible underlying mechanisms (Table 2).

**TABLE 2:** Modulators of microglia phenotypic transition.

**Modulators**		**M1/M2 ratio**	**Mechanism or pathway**	**References**
**Classification**	**Name**			
**Cytokines**	IL-6	Increase	Activation of the JAK1/2-STAT1/3 pathway	Burton et al., 2013; Haddick et al., 2017; Narazaki and Kishimoto, 2018
	IL-13	Decrease	Activation of the JAK-STAT6 pathway	Cherry et al., 2014; Junttila, 2018; Kolosowska et al., 2019; Quarta et al., 2019
	IL-4	Decrease	Activation of the JAK-STAT6 pathway	Beers et al., 2011; Gadani et al., 2012; Chhor et al., 2013; Aratake et al., 2018; Junttila, 2018
	IFN	Increase	Activation of the JAK1/2-STAT1 pathway	Boehm et al., 1997; Milner and Campbell, 2003; Kawanokuchi et al., 2004; de Weerd and Nguyen, 2012; Baer et al., 2016
**Ion channels**	ATP-sensitive potassium channel (Kir6.1)	Decrease	Inhibition of the p38-MAPK-NF-κB pathway	Ren et al., 2016; Du et al., 2018
	Voltage-gated potassium channel (Kv1.3)	Increase	Activation of the NLRP3 inflammasome	Chen Y. J. et al., 2018; Di Lucente et al., 2018; Maezawa et al., 2018; Ma et al., 2020; Sarkar et al., 2020
	Voltage-gated calcium channel (Cav1.2)	Increase	Up-regulation of the expression of Cacna1c, intracellular Ca2+, and pro-inflammatory factors	Espinosa-Parrilla et al., 2015; Wang et al., 2019
	Voltage gated proton channel (Hv1)	Increase	Down-regulation of the intracellular H+ which assists NADPH oxidase to generate ROS	Tian et al., 2016; Zhang Z. J. et al., 2020
**Receptors**	TREM2	Decrease	–	Zhang Y. et al., 2018
	CX3CR1	Increase	CX3CL1/CX3CR1 axis	Chapman et al., 2000; Zhang J. et al., 2018
**Oxidative stress reaction**	NADPH oxidase	Increase	Promotion of the ROS production	Rodriguez-Pallares et al., 2008; Rodriguez-Perez et al., 2015; Labandeira-Garcia et al., 2017
	ROS	Increase	–	Kwon et al., 2016; Sun H. et al., 2018; Ren et al., 2020
**Non-encoding RNA**	miR-155	Increase	Down-regulation of the expression of SOCS1	Butovsky et al., 2015; Sun X. et al., 2018; Fu et al., 2019
	miR-101	Increase	Down-regulation of the expression of MAPK phosphatase-1	Saika et al., 2017
	miR-125b	Increase	–	Cunha et al., 2018
	miR-146a	Increase	–	Cunha et al., 2018
	miR-21	Increase	–	Cunha et al., 2018
	miR-124	Decrease	Down-regulation of the expression of C/EBP-α; Down-regulation of the expression of P62 and P38	Yu et al., 2017; Yao et al., 2019
	miR-128	Decrease	Activation of the P38	Yang et al., 2017

*C/EBP-α, CCAAT/enhancer-binding protein-α; ROS, reactive oxygen species; SOCS1, suppressor of cytokine signaling 1.*

### Cytokines

#### Interleukin

Interleukin-6 (IL-6) is a kind of pro-inflammatory cytokine, which can be produced by a variety of CNS resident cells. Specifically, IL-6 plays an important role in the regulation of microglia polarization because the interleukin-6 receptor (IL-6R) required for the response exists only on the surface of microglia (Erta et al., 2012; Hsu et al., 2015). IL-6 binds to the membrane receptor IL-6R and induces homodimerization of gp130 to form the IL-6 signal complex (Narazaki and Kishimoto, 2018). In addition, IL-6 has a trans-signaling activation mode, which binds soluble IL-6R (sIL-6R) and activates gp130 on cells that do not express membrane-bound IL-6R on their surfaces (Rose-John, 2015). The intracellular signaling of IL-6 is initiated by intracellular tyrosine phosphorylation of gp130, which activates the downstream Janus family kinases (JAKs: JAK1, JAK2, and TYK2). STAT1 and STAT3, activated by JAK1 and JAK2, are transferred from the cytoplasm to the nucleus so as to regulate transcription (Narazaki and Kishimoto, 2018). Lateral intraventricular injection of soluble gp130 (sgp130), the inhibitor of sIL-6R, can reduce microglia-induced neuroinflammation in LPS-treated mice (Burton et al., 2013). Mutation of D358A allele of IL-6R leads to increased sIL6R release and increased neuroinflammation caused by the downstream genes of IL-6, which is associated with the early onset of AD (Haddick et al., 2017).

IL-13 and IL-4 are anti-inflammatory cytokines that promote the polarization of M2 microglia. IL-13 is secreted by Th2 cells and 30% sequence of IL-13 is identified with IL-4. Therefore, IL-13 and IL-4 share a common receptor subunit and both perform signal transduction through the JAK-STAT6 pathway (McCormick and Heller, 2015; Junttila, 2018). The expression of anti-inflammatory markers including Arg-1 and Ym1 is upregulated in IL-13-treated microglia (Cherry et al., 2014; Kolosowska et al., 2019; Quarta et al., 2019). IL-4 is secreted by Treg cells and Th2 cells, which upregulates the expression of M2-type markers in microglia, including Arginase-1, Ym1, Fizz1 and CD206 (Beers et al., 2011; Gadani et al., 2012; Chhor et al., 2013; Aratake et al., 2018). Inspiringly, intraperitoneal injection of IL-4 can regulate the phenotype of microglia after epilepsy, which might be explained by two potential mechanisms: one is the permeability of the blood-brain barrier changes after epilepsy, allowing IL-4 to enter the brain and play a role; the other one is that IL-4 regulates the peripheral mononuclear system and thus affects the microglia in the brain (Ravizza et al., 2008; Villa et al., 2016). Intracerebral injection of IL-4 can inhibit the polarization of M1 microglia and promote M2 microglia, thereby improving the recovery of neurological dysfunction after intracerebral hemorrhage (Yang et al., 2016). IL-4 treatment can also regulate microglia activation, reduce the release of pro-inflammatory cytokines, and improve the long-term harmful effects of seizures in the pilocarpine-induced epilepsy model (Li et al., 2017).

#### Interferon

Type I interferon is a subgroup of interferon proteins, including interferon-α (IFN-α) and interferon-β (IFN-β). Compared with astrocytes, microglia have broader and more diverse responses to type I interferons, so microglia are the main responsive cells of type I interferon in the CNS (Li et al., 2018). Type I interferon binds to IFNAR1 and IFNAR2, and activates IFNAR1 and IFNAR2 into a dimeric transmembrane complex. Then IFNAR1 and IFNR2 bind to TYK2 and JAK1, respectively, and activate the STAT signaling pathway (de Weerd and Nguyen, 2012).

Microglia exhibit amoeboid morphology, which usually represents an activated state in the presence of IFN-α (Milner and Campbell, 2003). IFN-β upregulates the expression of pro-inflammatory mediators such as TNFα, IL-1β, IL-6, and NO in LPS-stimulated microglia. It can also upregulate the expression of TNF-α and iNOS in unstimulated microglia (Kawanokuchi et al., 2004). The sustained stress response of damaged neurons caused by traumatic brain injury (TBI) increases IFN-β secretion, activates M1 microglia through STAT1 signaling pathway, and strengthens neuroinflammation (Sen et al., 2020). Compared with wild-type mice, IFN-β-deficient mice show a phenotype of decreased expression of pro-inflammatory mediators, decreased long-term microglia activation, decreased post-traumatic neuroinflammation, and improved neurological function recovery after TBI (Barrett et al., 2020). The expression of TNFα, IL-1β, and IL-6 are downregulated in IFNAR1-deficient mice, while the expression of IL-10 is upregulated after TBI, suggesting that inhibition of type I interferon can reduce post-TBI neuroinflammation (Karve et al., 2016). In addition, the microglia surrounding amyloid plaques are less reactive and exhibit an anti-inflammatory phenotype in IFNAR1-deficient mice (Minter et al., 2016). However, there are different opinions on the role of IFN-β in neuroinflammation. IFN-β can inhibit the release of glutamate and superoxide from activated microglia, protect neurons from neurotoxicity induced by microglia, and alleviate the neuroinflammatory response of MS (Jin et al., 2007). Increasing the content of IFN-β in the CNS can downregulate the activation of microglia and suppress neuroinflammation in experimental autoimmune encephalitis (EAE) mice (Gonzalez et al., 2021). Soluble IFN receptor (sIFNAR) enhances the immunomodulatory role of exogenous IFN-β in EAE by reducing the neuroinflammation induced by microglial activation (Suardiaz et al., 2016).

IFN-γ belongs to type II interferon. After binding to IFN-γ receptor (IFN-γR), IFN-γ regulates the expression of M1-type markers such as iNOS, TNF-α, IL-6 and IL-1β through JAK1/2-STAT1 signal pathway (Boehm et al., 1997; Kotenko and Pestka, 2000). IFN-γ induces microglia to polarize into a pro-inflammatory and cytotoxic M1 phenotype *in vitro* (Baer et al., 2016). Co-culture of Aβ and IFN-γ increases the expression of TNF-α and iNOS in microglia, while JAK2 inhibitor TG101209 decreases the expression of M1-type markers (Jones et al., 2015). IFN-γ can reduce the proportion of M2 microglia and the expression of IL-4 and IL-10 after epilepsy (Li et al., 2017). However, high expression of M1 microglia-related markers is also inhibited, which may be related to the negative feedback of IFN-γ induced inflammatory response with upregulation of SOCS3 (Boontanrart et al., 2016; Li et al., 2017).

### Ion Channels

#### ATP-Sensitive Potassium Channel

ATP-sensitive potassium channel (K-ATP) is a unique octahedron channel that couples cell metabolism and membrane potential, which is comprised of Kir6.x (6.1 or 6.2) subunits that form pores and sulfonylurea receptors (SUR1 or SUR2) regulated by intracellular ATP and ADP levels (Rubaiy, 2016). This channel stabilizes the resting membrane potential; moreover, blocking it depolarizes cells and reduces the driving force of Ca^2+^ influx into macrophages and microglia (Tsai et al., 2013). As a metabolic sensor, the K-ATP channel is widely expressed in most metabolically active tissues, including the brain. Kir6.1 is mainly expressed in microglia and astrocytes; further, it is involved in neuroinflammation (Thomzig et al., 2001; Zhou et al., 2008; Sun and Hu, 2010; Fan et al., 2016).

Kir6.1 inhibition suppresses M2 microglial polarization and promotes M1 type inflammatory response; contrastingly, Kir6.1 overexpression promotes M2 microglial polarization and reduces M1 type toxicity (Du et al., 2018). *Kir6.1* knockout mouse established using 1-methyl-4-phenyl-1,2,3,6-tetrahydropyridine (MPTP) can increase the ratio of M1/M2 microglia and aggravate dopaminergic neuronal death by activating the p38/MAPK/NF-κB pathway in microglia in the substantia nigra pars compacta (Du et al., 2018). Additionally, CD200 can promote K-ATP channel opening, which subsequently inhibits microglia activation and pro-inflammatory cytokine release, protects dopaminergic neurons, and alleviates PD symptoms (Ren et al., 2016). Taken together, Kir6.1 channel activation can transit microglia to the M2 phenotype and exert protective effects.

#### Voltage-Gated Potassium Channel

The voltage-gated potassium channel Kv1.3 is a transmembrane protein comprised of four pore-forming α-subunits and four auxiliary β-subunits. It affects the inflammatory response through its involvement in the production of inflammatory cytokines and ROS; moreover, it is expressed in microglia (Gonzalez et al., 2012; Serrano-Albarras et al., 2018; Sarkar et al., 2020). Blocking Kv1.3 promotes M2 microglia polarization and inhibits M1 microglia polarization in various animal models of neurodegenerative diseases (Rangaraju et al., 2018; Sarkar et al., 2020). Therefore, Kv1.3 can be used as a potential neuroimmune regulatory target.

LPS alone or with IFN-γ induce microglial transition into the M1 phenotype with a higher Kv1.3 current density. Contrastingly, PAP-1, a small-molecule Kv1.3 inhibitor, and SHK-18631, a peptide inhibitor, can significantly reduce pro-inflammatory cytokine expression and NO release during M1 microglial polarization (Nguyen et al., 2017). The Kv1.3 channel is highly expressed on activated M1 microglia in middle cerebral artery occlusion/reperfusion (MCAO/R) mice, which is a common stroke animal model. Blocking Kv1.3 using PAP-1 can reduce M1 microglia polarization, inhibit inflammation, and reduce the infarcted brain tissue volume (Chen Y. J. et al., 2018). Using PAP-1 to block Kv1.3 may decrease the ratio of M1/M2 microglia, which alleviates inflammation and damage in MCAO/R rats and oxygen-glucose deprivation/reperfusion microglia. The underlying mechanism may involve NLRP3 inflammasome inhibition in microglia, which subsequently decreases IL-1β release (Ma et al., 2020). Kv1.3 is highly expressed in microglia of the AD mouse model and human AD brain slices. PAP-1 can reduce pro-inflammatory cytokine expression and neurotoxicity in microglia induced by amyloid β oligomers (Maezawa et al., 2018). Further, Kv1.3 gene knockout can eliminate LPS-induced microglial activation characterized by Iba-1 immune response, as well as eliminate the expression of pro-inflammatory factors including IL-1β, tumor necrosis factor-α (TNF-α), IL-6, and iNOS (Di Lucente et al., 2018). In summary, Kv1.3 inhibitors can be used to inhibit the harmful neuroinflammatory response induced by M1 microglial polarization.

#### Voltage-Gated Calcium Channel

The voltage-gated calcium channel Cav1.2 is a transmembrane channel protein comprised of α1, α2/δ, and β subunits. The α1 subunit is comprised of conduction pores, voltage sensors, and gating devices; moreover, it is the site of multiple second messengers, drugs, and toxins (Catterall et al., 2005). Cav1.2 is expressed in excitable cells and microglia (Espinosa-Parrilla et al., 2015).

Calcium antagonists can reduce microglial production of TNF-α and NO, as well as reduce neurotoxicity; however, it does not significantly alter the microglia phagocytic activity in a rat model of *N*-methyl-D-aspartate-induced hippocampal neurodegeneration (Espinosa-Parrilla et al., 2015). Contrastingly, another study reported that microglia activated by IL-4 treatment with a calcium antagonist showed reduced arginase 1 expression, which is indicative of reduced M2 phenotype transition. On the other hand, MG6 cells were stimulated by low LPS/IFN-γ levels, which increased the expression of Cacna1c encoding Cav1.2, intracellular Ca^2+^ levels, and pro-inflammatory factor expression. Microglia activated through LPS treatment with nifedipine, which is a calcium antagonist, promoted iNOS expression in M1 microglia. However, high levels of a calcium antagonist inhibited M1 phenotype activation (Wang et al., 2019). Severe dopamine neuronal degeneration was accompanied by significant behavioral defects in Cav1.2 knockout mice with MPTP (Wang et al., 2019). Although these findings are inconsistent, they indicate that Cav1.2 is crucially involved in microglial phenotypic transition.

#### Voltage-Gated Proton Channel

The voltage-gated proton channel Hv1 is a dimer channel protein, with each subunit containing a channel and voltage sensor; consequently, Hv1 has two independent conduction pathways, which are highly synergistic upon activation (Qiu et al., 2013). Although Hv1 is not expressed in mouse hippocampal pyramidal neurons and astrocytes, it is selectively and functionally expressed in microglia (Wu, 2014).

Upon microglia depolarization, Hv1 mediates intracellular H^+^ outflow, and therefore regulates intracellular pH and facilitates ROS generation mediated by NADPH oxidase (NOX) (Wu, 2014). Compared with wild-type mice, Hv1 knockout mice had microglia that tended to transit to the protective M2 phenotype, with decreased iNOS and CD16 expression; increased CD26 and arginase expression; and a reduced incidence of cerebral infarction, neuronal damage, and movement disorders in ischemic stroke (Tian et al., 2016). Moreover, age is associated with Hv1 channel expression. Compared with adult mice (2–3 months), elderly mice (18 months) have shown significantly increased Hv1 expression, with a tendency of polarization toward the pro-inflammatory M1 phenotype and reduced anti-inflammatory M2 polarization (Zhang Z. J. et al., 2020). In summary, Hv1 channel blocking promotes microglial transition to the M2 phenotype, and therefore is a potential therapeutic target for neuroinflammation.

### Receptors

#### TREM2

Trigger receptor expressed on myeloid cells 2 (TREM2) is a transmembrane receptor exclusively expressed on microglia, which adjusts microglia function by regulating microglia activation (Frank et al., 2008; Kawabori et al., 2015). TREM2 is a protective factor for neurodegenerative diseases; moreover, TREM2 mutations are associated with an increased PD risk (Rayaprolu et al., 2013; Jiang et al., 2014, 2016). The expression level of TREM2 level influence the ratio of M1/M2 microglia. TREM2 gene knockout inhibits M2 microglial polarization and increases the inflammatory response of M1 microglia. On the other hand, TREM2 overexpression can promote M2 microglial polarization and reduce microglia-induced inflammatory response (Zhang Y. et al., 2018).

#### CX3CR1

Neurons express chemokine C-X3-C motif ligand 1 (CX3CL1), which mediates microglial activation by interacting with the unique receptor CX3CR1 on microglia (Chapman et al., 2000). An *in vivo* study reported significantly increased CX3CL1 expression in motor neurons in the anterior horn of the spinal cord in SOD1^*G93A*^ mice, which is a model of amyotrophic lateral sclerosis (ALS), at 40 days; however, it decreased at 90 and 120 days due to motor neurons loss in the anterior horn. The CX3CR1 mRNA level in the anterior horn region did not significantly increase at 40 days; however, it increased at 90 and 120 days, which indicated obvious microglial activation in the anterior horn. However, at 120 days, there was an increase and decrease in M1-related and M2-related cytokines, respectively (Zhang J. et al., 2018). Moreover, protein expression analysis of SOD 1^*G93A*^ mice during the symptomatic phase revealed significant upregulation of CX3CL1-CX3CR1 axis expression (Cunha et al., 2018). Given the dynamic changes of the CX3CL1/CX3CR1 axis and the unbalanced M1/M2 microglia activation in the pathological process of ALS, elucidation of the molecular mechanism through which the CX3CL1/CX3CR1 axis regulates microglial activation may render the CX3CL1/CX3CR1 axis as a therapeutic target for ALS.

### Oxidative Stress Reaction

#### NADPH Oxidase

NOX is a superoxide dismutase that is highly expressed in microglia (Hou et al., 2018). NOX activation can produce both extracellular and intracellular ROS, which are crucially involved in mediating chronic neuroinflammatory responses and associated neuronal injury (Zhang C. et al., 2018). Angiotensin II is the main activator of the NOX complex in microglia. It enhances NOX complex activity through angiotensin type I (AT1) receptor, promotes ROS production, and mediates microglial polarization to M1, which subsequently enhances neuroinflammation (Rodriguez-Pallares et al., 2008; Rodriguez-Perez et al., 2015; Labandeira-Garcia et al., 2017).

AT1 activation intensifies the microglial inflammatory response, oxidative stress, and dopaminergic degeneration; moreover, AT1 receptor blockers have inhibitory effects on 6-hydroxydopamine and MPTP-induced PD models (Rey et al., 2007; Rodriguez-Pallares et al., 2008; Joglar et al., 2009). Overexpression of α-syNuclein in dopaminergic neurons can induce AT1 expression and increased NOX activity. AT1 receptor blockers can inhibit α-syNuclein-induced microglial phenotype change and dopaminergic neuronal death, which induces M2 microglia polarization and plays a neuroprotective role (Rodriguez-Perez et al., 2018). Apocynin and Taurine significantly improved learning and memory deficits in mice by reducing NOX activation and related oxidative stress, as well as inhibiting M1 microglial polarization; moreover, they alleviated neuroinflammation and nerve injury in the paraquat and maneb-induced PD model by inhibiting the STAT1 and NF-κB pathway (Che et al., 2018; Hou et al., 2019).

#### Reactive Oxygen Species

Mitochondria are the main production sites of ROS, which can induce M2-to-M1 phenotypic transition and cause AD (Kwon et al., 2016; Sun H. et al., 2018). The mitochondrial-targeting TPP-MoS2 QDs are nano-enzymes with the dual enzymatic activities of superoxide dismutase (SOD) and catalase, which employ MoS2 nanoparticles to eliminate ROS generated by mitochondrial oxidative stress. On the other hand, triphenyl-phosphonium bromide (TPP) is a lipophilic cation that targets nanozymes to mitochondria through negative mitochondrial membrane potential (Marrache and Dhar, 2012; Zhang et al., 2016; Yim et al., 2018). TPP-MoS2 QDs can cross the blood-brain barrier; remove mitochondrial produced ROS; suppress the expression of pro-inflammatory cytokines IL-1β, IL-6, and TNF-α; promote M2 microglial polarization; and play a neuroprotective role. Moreover, TPP-MoS2 QDs can reduce toxicity by clearing hippocampal Aβ deposition in AD mice (Ren et al., 2020).

### MicroRNAs

MicroRNAs (miRNAs) are endogenous non-coding RNAs that affect gene expression through mRNA regulation. Studies have reported various miRNAs involved in microglial polarization. For example, M1 microglia showed increased expression of miR-155, miR-101, miR-125b, miR-146a, and miR-21, which may be associated with microglial transition to the pro-inflammatory phenotype. On the other hand, there is increased expression of miR-124 and miR-128 in M2 microglia, which may be associated with microglial transition to the anti-inflammatory phenotype.

#### MicroRNAs Promoting M1 Polarization

It has been reported that miR-155 is crucially involved in inflammatory reactions, with overexpression in SOD1^*G93A*^ mice, as well as in human patients with sporadic and familial ALS. Anti-miR-155 treatment can reduce M1 microglial polarization and relieve ALS symptoms (Butovsky et al., 2015). There was significantly increased cortical and hippocampal miR-155 expression in a kainic acid-induced epileptic mouse model. Further treatment with miR-155 antagonist decreased the expression of inflammatory cytokines IL-1β and IL-6, as well as reversed morphological changes in microglia (Fu et al., 2019). Inhibiting miR-155 can promote microglial transition to an anti-inflammatory phenotype by increasing SOCS1 expression in the BV2 microglial cell line (Sun X. et al., 2018). Additionally, miR-101 can promote microglial expression of pro-inflammatory cytokines, including IL-6 and TNF-α (Saika et al., 2017). An *in vitro* study reported that exosomes released by NSC-34 motoneuron-like cells transfected with mutant SOD (mSOD) are rich in miR-124 and preferentially internalized using N9 microglia, which results in microglial activation, as well as the release of various pro-inflammatory mediators and cytokines (Pinto et al., 2017). During the symptomatic stage in SOD1^*G93A*^ mice, there is significant upregulation of inflammation-related miRs, including miR-125b, miR-146a, and miR-21 (Cunha et al., 2018). Therefore, inflammation-related miRs may be extensively involved in microglial phenotypic transitions.

#### MicroRNAs Promote M2 Polarization

miR-124 inhibits C/EBP-α expression by binding to the 3′ untranslated regions of C/EBP-α, which downregulates IL-6, IL-1β, and TNF-α expression; promotes microglial transition to the M2 phenotype; and alleviates inflammatory injury caused by intracerebral hemorrhage (Yu et al., 2017). Contrastingly, miR-124 knockdown significantly decreases TNF-α and IL-1β levels in LPS-induced BV2 microglial cell lines (Yao et al., 2019). MiR-128 can downregulate the expression of M1 microglia markers CD86 and CD32; upregulate the expression of M2 microglia marker CD206; promote M2 microglial polarization; and downregulate TNF-α, IL-1β, and IL-6 expression (Yang et al., 2017).

**FIGURE 2:**
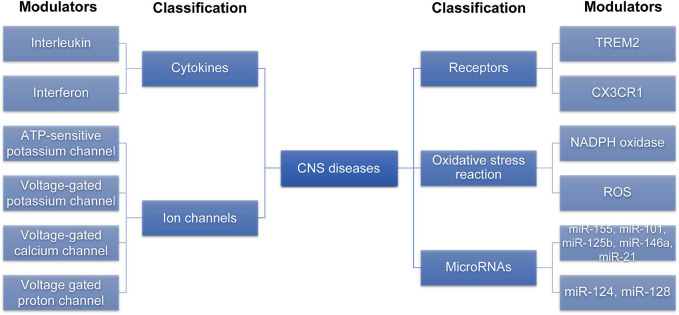
The modulators involved in the microglia phenotypic transition. Various modulators can regulate M1/M2 microglia phenotypic transition. This figure is summarized by classification of different modulators. CNS, central nervous system; ROS, reactive oxygen species; TREM2, trigger receptor expressed on myeloid cells 2; CX3CR1, C-X3-C motif ligand 1 receptor.

After summarizing the modulators that may be involved in the of microglial phenotypic transition (Figure 2), we summarized the common signaling pathways that these modulators play a role in, providing a reference for us to comprehensively understand the role of these modulators in regulating the microglial phenotypic transition.

## Signal Pathways Involved in Regulating Microglial Phenotypic Transition

**FIGURE 3:**
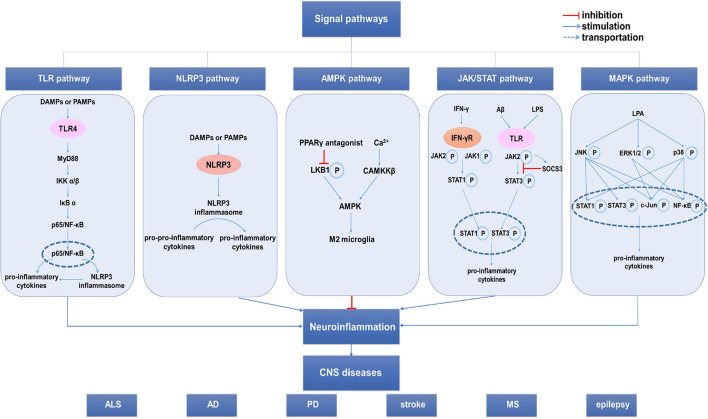
The signal pathways involved in the microglia phenotypic transition. Several signal pathways are involved in the regulation of the microglia phenotypic transformation, mainly including the TLR4/NF-κB pathway, NLR pathway and JAK/STAT pathway. Additionally, AMPK pathway and MAPK pathway can also affect the microglia phenotypic transition. These pathways influence neuroinflammation by regulating the microglia phenotypic transition, thus influencing the occurrence, development, and prognosis of CNS diseases. AD, Alzheimer’s disease; ALS, amyotrophic lateral sclerosis; CNS, central nervous system; MS, multiple sclerosis; NLR, NOD-like receptor; PD, Parkinson’s disease; SOCS3, suppressor of cytokine signaling 3; TLR, Toll-like receptor.

Numerous signaling pathways, including inflammation- and cytokine-related signaling pathways, are involved in microglial phenotypic transition. For the sake of brevity, this review focuses on the more classical signaling pathways (Figure 3) while briefly summarizing the other signaling pathways.

### TLR4/NF-κB Pathway

TLR4/NF-κB is a classic inflammation-related signaling pathway involved in regulating microglial phenotypic polarization; moreover, activating this signaling pathway promotes M1 microglial polarization (Saijo and Glass, 2011). Upon neuronal damage and death, there is release of endogenous damage-associated molecular patterns (DAMPs), which activate TLR4 on the microglia surface, resulting in focal neuroinflammation (Eldahshan et al., 2019; Kumar, 2019). Furthermore, exogenous pathogen-associated molecular patterns (PAMPs), including LPS, are classic microglia-stimulating factors that activate TLR4 on the microglia surface to produce pro-inflammatory cytokines, including TNF-α and IL-6 (Kuroda et al., 2020). In conclusion, after CNS damage, DAMPs and PAMPs can act on TLR4 to activate microglia and promote pro-inflammatory effects. Interventions on the TLR4/NF-κB signaling pathway can regulate microglial phenotypic transition, and therefore reduces neuroinflammation and neuronal injury.

TAK-242, which is a TLR4 specific inhibitor, induces microglial polarization to the M2 phenotype and exerts a neuroprotective effect on AD mice, with the underlying mechanism possibly being related to the TLR4/MyD88/NF-κB/NLRP3 signaling pathway (Cui et al., 2020). In the TBI model, *TLR4* knockout mice have more M2 phenotype microglia than wild-type mice, which ameliorates TBI-induced neurological dysfunction (Yao et al., 2017). Omega-3 polyunsaturated fatty acids suppress subsequent inflammatory reactions by reducing NF-κB pathway activation after TBI-induced microglial activation (Chen X. et al., 2018). Taurine protects dopaminergic neurons in PD mice by inhibiting NF-κB signaling pathway activation, as well as reducing microglial M1 polarization and pro-inflammatory mediator expression (Che et al., 2018). Furthermore, some common drugs can regulate microglial phenotype transition by inhibiting the TLR4/NF-κB signaling pathway. Specifically, candesartan converts microglia to the M2 phenotype, which reduces stroke-induced neuronal injury by inhibiting TLR4 expression, IKBα and p65 phosphorylation, and NF-κB/p65 nuclear translocation in a TLR4/NF-κB dependent mechanism (Qie et al., 2020). Pinocembrinin decreases the expression of TLR4 and its downstream target proteins, TRIF and MyD88, which reduces M1 microglial polarization and nerve injury after cerebral hemorrhage; however, this protective effect is absent in mice without microglia and *TLR4* knockout mice (Lan et al., 2017). Meisoindigo promotes M1-to-M2 microglial phenotype transition by inhibiting the TLR4/NF-κB signaling pathway, which effectively prevents focal cerebral ischemia-reperfusion injury (Ye et al., 2019). Fasudil inhibits the expression of TLR4, MyD88, and NF-κB; moreover, it promotes microglia transition to the M2 phenotype in AD mice through the TLR4/MyD88/NF-κB pathway (Guo et al., 2020). Contrastingly, activating the TLR4/NF-κB signaling pathway promotes microglial M1 polarization and aggravates nerve injury (Chen et al., 2019). Cathepsin C promotes microglial M1 polarization and exacerbates neuroinflammation by activating the NF-κB signaling pathway (Liu et al., 2019). Paraquat upregulates TLR4 and MyD88 expression and promotes NF-κB/p65 translocation; moreover, it promotes transition to the M1 phenotype and secretion of inflammatory mediators, including TNF-α, IL-1β, and IL-6 by activating the TLR4/NF-κB signaling pathway (Huang et al., 2019).

In conclusion, inhibiting the TLR4/NF-κB signaling pathway effectively reduces the polarization of M1-type microglia in various CNS diseases, and therefore reduces pro-inflammatory mediator secretion and neuroinflammation, which exerts neuroprotective effects. Contrastingly, activating the TLR4/NF-κB signaling pathway may aggravate nerve injury.

### NLR Pathway

NOD-like receptor pyrin-domain-containing 3 (NLRP3) belongs to the NOD-like receptor (NLR) family, which is activated by PAMPs and DAMPs to form a polymer protein complex known as NLRP3 inflammasome. The NLRP3 inflammasome induces the release of pro-inflammatory cytokines IL-1β and IL-18; moreover, it is considered a major player in neuroinflammation (Sui et al., 2020; Zhao Z. et al., 2020). Currently, there is extensive research on neuroinflammation inhibition by targeting the NLRP3 inflammasome (Cui et al., 2020; Wang et al., 2020). NLRP3-mediated neuroinflammation is closely associated with microglial activation and polarization. *In vivo* and *in vitro* experiments have demonstrated that inhibiting the activation of NLRP3 inflammasome can promote M1-to-M2 microglial phenotype transition, alleviate post-stroke neuroinflammation, reduce neuronal injury, improve cognitive impairment, and reduce cerebral ischemia-reperfusion injury (Ye et al., 2019; Du et al., 2020; Sui et al., 2020; Zhao J. et al., 2020; Cao et al., 2021; Ma et al., 2021). Inhibiting NLRP3 inflammasome-mediated neuroinflammation is a potential treatment target for cerebral hemorrhage (Chen et al., 2020; Xiao et al., 2020). Inhibiting the TLR4/NF-κB/NLRP3 signaling pathway can induce M2 microglial polarization in patients with AD, which reduces neuroinflammation and improves learning and memory disorders (Cui et al., 2020; Yan et al., 2020). Other than classic NLRP3, inhibiting neuroinflammation mediated by other NLR family members, including absent in melanoma 2, can also effectively reduce post-TBI nerve injury (Sun et al., 2020).

### JAK/STAT Pathway

The Janus kinase/signal transducer and activator of transcription (JAK/STAT) signaling pathway regulates microglial phenotypic transition by influencing cytokine secretion, and therefore regulates neuroinflammation. Specifically, there has been more extensive research on the JAK2/STAT3 signaling pathway (Jones et al., 2015; Iwahara et al., 2017). The suppressor of cytokine signaling (SOCS) family negatively regulates the JAK/STAT pathway, which prevents long-term JAK/STAT pathway activation and is crucially involved in regulating the inflammatory response (Kuroda et al., 2020). In the resting state, neurons and glial cells express SOCS, which downregulates JAK/STAT signaling pathway activity and promotes microglial differentiation into an anti-inflammatory phenotype (Bedoui et al., 2018). In microglia, LPS rapidly activates the JAK2/STAT3 signaling pathway; however, SOCS1 expression upregulation can effectively block JAK2/STAT3 function and reduce the ratio of M1/M2 microglia (Porro et al., 2019). Additionally, Aβ stimulation induces microglia to produce a unique M1 phenotype in AD (TNF-α and SOCS are upregulated while IL-6 is not). In these microglia, there is a concomitant increase in SOCS3 and pro-inflammatory cytokine levels; therefore, SOCS3 is thought to inhibit microglial M1 polarization in AD by inhibiting the JAK2/STAT3 signaling pathway to block IL-6 production (Iwahara et al., 2017). SOCS1/3 expression can reduce the M1/M2 microglia ratio and alleviate post-TBI neuroinflammation (Ruganzu et al., 2021). Additionally, TREM2 overexpression in AD mice can attenuate the inflammatory response induced by M1 microglia through inhibition of the JAK2/STAT3 signaling pathway, which consequently attenuates neuroinflammation (Ruganzu et al., 2021). Inhibiting the JAK2-STAT3 pathway in early stage acute spinal cord injury regulates microglial phenotype transition, reduces inflammatory cytokine expression, alleviates neuronal apoptosis, and promotes functional recovery (Zheng et al., 2020). Moreover, inhibiting the JAK/STAT signaling pathway reduces the expression and release of inflammatory cytokines and chemokines in EAE mice, and therefore reduces neuroinflammation (Qu et al., 2019).

IFN-γ binds to the IFN-γ receptor and induces heterodimerization and phosphorylation of JAK 1 and JAK 2, which causes STAT generation and nucleus translocation, and therefore promotes IFN-γ expression (Kotenko and Pestka, 2000). IFN-γ regulates > 200 genes, including M1 microglial markers (Martinez and Gordon, 2014). Specifically, IFN-γ binds to the IFN-γ receptor to activate the JAK/STAT signaling pathway and promote M1 microglial polarization. JAK2 inhibitors can block the IFN-γ-induced increase of M1 cell markers *in vivo* and *in vitro* (Jones et al., 2015).

Generally, JAK/STAT signaling pathway activation causes M1 microglial polarization; therefore, inhibiting the JAK/STAT signaling pathway may be another potential strategy for treating neuroinflammation.

### Other Pathways

In addition to these classical signaling pathways, other pathways can directly or indirectly regulate microglial phenotypic transition by acting on the aforementioned signaling pathways. Activation of the AMP-activated protein kinase (AMPK) signaling pathway promotes post-stroke M2 microglia polarization and alleviates neuroinflammation (Wang et al., 2018; Zheng et al., 2019; Zhou et al., 2019). Curcumin activates the CaMKKβ-dependent AMPK signaling pathway, promotes microglial transition into the M2 type, and prevents neuroinflammation; moreover, it is considered a novel therapeutic agent for neurodegenerative diseases (Qiao et al., 2020). Mesenchymal stem cell-derived extracellular vesicles activate AMPK and inhibit the NF-κB signaling pathway, which promotes M2 microglial polarization to reduce post-SAH neuroinflammation (Han et al., 2021). Peroxisome proliferator-activated receptor γ (PPARγ) agonists activate AMPK and inhibit the NF-κB signaling pathway, which facilitates M1-to-M2 phenotype microglial transition (Ji et al., 2018).

Lysophosphatidic acid mediates M1 microglial polarization through the mitogen-activated protein kinase (MAPK)-dependent pathway, which can be reversed by MAPK pathway antagonists (Plastira et al., 2020). Dexmedetomidine inhibits ERK1/2 phosphorylation to block the MAPK/ERK signaling pathway, increases M2 microglial polarization, and exerts anti-inflammatory effects in BV2 cells (Qiu et al., 2020). SCP2-1, which is a natural product of a homogeneous polysaccharide from S. Chinensis, can reduce microglial activation and M1 polarization through NF-κB and MAPK pathway inhibition; moreover, it improves cognitive dysfunction (Xu et al., 2020).

## Conclusion and Foresight

Currently, numerous challenges are facing clinical treatment of CNS diseases with respect to effectiveness and safety. Numerous drugs cannot reach the brain tissue due to the blood-brain barrier. Neuroinflammation is a common feature of CNS diseases; therefore, regulating microglial phenotype transition has become a promising therapeutic strategy. As described here, numerous modulators affect microglial polarization. Moreover, altering the CNS immune microenvironment by inhibiting M1 microglia polarization and/or promoting M2 microglia polarization, respectively, which have corresponding neurotoxic and neuroprotective effects, respectively, could allow neuroprotection in various CNS diseases.

As previously mentioned, some specific microglia phenotypic transition modulators (e.g., iron channels, receptors, oxidative stress reaction-related substances, and microRNAs) summarized in this review have strong selectivity, so rational use of these modulators can effectively regulate neuroinflammation, and point out a clear direction for the treatment of neuroinflammation-related CNS diseases.

In addition, signaling pathways involved in regulating phenotypic transformation of microglia (e.g., TLR pathway, NLR pathway, and JAK/STAT pathway) may serve as potential targets for the regulation of neuroinflammation. All the blockers in these signaling pathways can act as modulators to regulate microglial cell transition to M2 phenotype and reduce neuroinflammation through this mechanism. However, if the signaling pathways of all cells are not selectively blocked, the normal physiological function of other cells may be affected. Therefore, how to target these signaling pathways in microglia cells is a problem worthy of further study.

In brief, this review is relevant in today’s scenario given the high prevalence and public health importance of CNS disorders and the need to develop safe and effective therapeutic strategies. Therefore, the findings of this study have strong implications. The findings have been discussed very well in terms of previous findings, and how the findings can be implemented. We make a significant novel contribution to the existing research because we provide molecular insights into the phenotypic microglial transition, which could be a potential therapeutic target for neuroinflammation and thus has important implications for drug development and clinical work.

## Author Contributions

JL and XS collected the literature material. RS, LW, and BZ prepared the picture and revised the manuscript. ZL designed the outline. BX provided the financial support. All the authors have approved the publication of the manuscript.

## Conflict of Interest

The authors declare that the research was conducted in the absence of any commercial or financial relationships that could be construed as a potential conflict of interest.

## Publisher’s Note

All claims expressed in this article are solely those of the authors and do not necessarily represent those of their affiliated organizations, or those of the publisher, the editors and the reviewers. Any product that may be evaluated in this article, or claim that may be made by its manufacturer, is not guaranteed or endorsed by the publisher.

## Abbreviations

Aβ: amyloid-β
AD: Alzheimer’s disease
ALS: amyotrophic lateral sclerosis
AMPK: AMP-activated protein kinase
AT1: angiotensin type I receptor
CNS: central nervous system
CX3CL1: C-X3-C motif ligand 1
DAMP: damage-associated molecular pattern
IFN-γ: interferon-γ
IL: interleukin
JAK/STAT: Janus kinase/signal transducer and activator of transcription
LPS: lipopolysaccharide
MAPK: mitogen-activated protein kinase
miRNA: microRNAs
MS: multiple sclerosis
NLR: NOD-like receptor
NLRP3: NOD-like receptor pyrin-domain-containing 3
NOX: NADPH oxidase
PAMP: pathogen-associated molecular pattern
PD: Parkinson’s disease
ROS: reactive oxygen species
SOCS: suppressor of cytokine signaling
SOD: superoxide dismutase
TBI: traumatic brain injury
TLR: toll-like receptor
TNF-α: tumor necrosis factor-α
TPP: triphenyl-phosphonium bromide
TREM2: trigger receptor expressed on myeloid cells 2.
